# Expression of catalase and retinoblastoma-related protein genes associates with cell death processes in Scots pine zygotic embryogenesis

**DOI:** 10.1186/s12870-015-0462-0

**Published:** 2015-03-15

**Authors:** Jaana Vuosku, Suvi Sutela, Johanna Kestilä, Anne Jokela, Tytti Sarjala, Hely Häggman

**Affiliations:** Genetics and Physiology Unit, University of Oulu, P.O. Box 3000, FI-90014 Oulu, Finland; Natural Resources Institute Finland (Luke), Parkano Unit, Kaironiementie 15, FI-39700 Parkano, Finland; Current address: Natural Resources Institute Finland (Luke), Rovaniemi Unit, FI-96301 Rovaniemi, Finland

**Keywords:** Autophagy, Catalase, Conifer, Developmental cell death, Embryogenesis, Megagametophyte, *Pinus*, Retinoblastoma-related protein, Scots pine, Seed development

## Abstract

**Background:**

The cell cycle and cellular oxidative stress responses are tightly controlled for proper growth and development of Scots pine (*Pinus sylvestris* L.) seed. Programmed cell death (PCD) is an integral part of the embryogenesis during which megagametophyte cells in the embryo surrounding region (ESR) and cells in the nucellar layers face death. In the present study, we show both the tissue and developmental stage specific expression of the genes encoding the autophagy related ATG5, catalase (CAT), and retinoblastoma related protein (RBR) as well as the connection between the gene expressions and cell death programs.

**Results:**

We found strong *CAT* expression in the cells of the developing embryo throughout the embryogenesis as well as in the cells of the megagametophyte and the nucellar layers at the early embryogeny. The *CAT* expression was found to overlap with both the *ATG5* expression and hydrogen peroxide localization. At the late embryogeny, *CAT* expression diminished in the dying cells of the nucellar layers as well as in megagametophyte cells, showing the first signs of incipient cell death. Accumulation of starch and minor *RBR* expression were characteristic of megagametophyte cells in the ESR, whereas strong *RBR* expression was found in the cells of the nucellar layers at the late embryogeny.

**Conclusions:**

Our results suggest that ATG5, CAT, and RBR are involved in the Scots pine embryogenesis and cell death processes. CAT seems to protect cells against hydrogen peroxide accumulation and oxidative stress related cell death especially during active metabolism. The opposite expression of *RBR* in the ESR and nucellar layers alongside morphological characteristics emphasizes the different type of the cell death processes in these tissues. Furthermore, the changes in *ATG5* and *RBR* expressions specifically in the megagametophyte cells dying by necrotic cell death suggest the genetic regulation of developmental necrosis in Scots pine embryogenesis.

**Electronic supplementary material:**

The online version of this article (doi:10.1186/s12870-015-0462-0) contains supplementary material, which is available to authorized users.

## Background

In multicellular organisms such as plants, the organized destruction of cells by programmed cell death (PCD) is essential for body plans and specific organ shapes as well as for removing damaged or infected cells [1-4]. In plants, different cell death types are often defined by morphological characteristics because precise molecular mechanisms behind the regulation and execution of cell death processes are still poorly known [5,6]. Two classes of cell death, vacuolar cell death and necrosis, have been suggested for plants but the terms are not unambiguous [6,7]. Examples of vacuolar cell death are found during embryo, organ and tissue morphogenesis and senescence, whereas necrosis is most usually induced by a variety of abiotic stresses and by successful recognition of a pathogen during the hypersensitive response [6,8]. However, necrosis is no longer considered to be an uncontrolled process, but necrotic cell death can also be a regulated event that contributes to the development and to the maintenance of tissue and organismal integrity [9-11]. In plants, the roles of nucleases and proteases in PCD are evident [12,13] but the cell death processes also involve many other molecular mechanisms such as catalase [14] and the retinoblastoma related proteins [15] which are required to maintain cellular homeostasis and thus may be difficult to identify as actual cell death effectors.

The reactive oxygen species (ROS) contribute to the induction, signalling and execution of plant cell death [16-18]. Hydrogen peroxide (H_2_O_2_), the most stable ROS [19], has received particular attention as a signal molecule involved in the regulation of essential biological functions such as cell death, pathogen responses, and gene expression [16,20-23]. H_2_O_2_ is a toxic byproduct of cell metabolism and H_2_O_2_ generation from a variety of cellular processes increases in response to numerous developmental signals and biotic and abiotic stimulants [24]. While plants contain several types of H_2_O_2_-metabolizing proteins, catalases (CAT, hydrogen peroxide oxidoreductase, EC 1.11.1.6) are highly active peroxisomal enzymes which express high specificity towards H_2_O_2_ and protect cells from the toxic effects by the conversion of H_2_O_2_ into water and molecular oxygen [25]. It has been suggested that impact of H_2_O_2_ is strongly influenced by the extent of accumulation of H_2_O_2_ allowed by antioxidant enzymes such as CAT. This has been shown in *Chlamydomonas reinhardtii* where a reversible partial inactivation of CAT correlates with a transient increase in the level of H_2_O_2_. The concentration range seems to be necessary to activate H_2_O_2_-dependent signaling pathways stimulating the expression of H_2_O_2_ responsive genes [26]. Furthermore, the direct interaction of CAT with ROS could allow CAT to act as a molecular link between ROS and the promotion of autophagy-dependent cell death [14].

In plants, the retinoblastoma related proteins (RBR) regulate the progression of cell cycle and transcription via chromatin-modifiers and, furthermore, function in promotion of cell differentiation [27]. Recently, RBR1 was found to control also cell death in maize (*Zea mays* L.) endosperm cells [15]. The plant RBR proteins are homologs of retinoblastoma susceptibility gene products (pRB) firstly discovered in human cells [28]. The pRB proteins are activated by phosphorylation and their A/B pocket domain enables them to interact with more than 100 proteins [29]. The interaction of hypophosphorylated RBR with E2F and DP transcription factors prevents cell division by suppressing the transition from G1 to S phase in the cell cycle [27]. In plants, *RBR* genes are conserved and they have been found from green algae, bryophytes, lycophytes and from both monocot and dicot angiosperm species [27,30]. In our previous study, we showed that the RBR/E2F pathway operates also in conifers by presenting the expression of *RBR* and *E2F* genes in *in vitro* cultured Scots pine (*Pinus sylvestris* L.) embryogenic cells [31].

PCD is an integral part of the seed development in angiosperm and gymnosperm species (e.g. [32-35]). In gymnosperms, the Scots pine seed represents a well-documented embryogenesis with coordinated action of several distinct cell death programs. The fertilization of several eggs [36] and furthermore the cleavage of polyzygotic embryos [37] leads to the presence of several embryos in a young seed. However, only the dominant embryo survives, whereas the subordinate embryos die via PCD during the seed development [34]. Embryos grow and develop inside the corrosion cavity of the megagametophyte tissue which functions as a nutrient source of embryos i.e. being congruent with the endosperm of angiosperms [38]. Throughout the embryo development, the megagametophyte cells in the embryo surrounding region (ESR) are destroyed by morphologically necrotic cell death [35]. Additionally, the cells in the megagametophyte surrounding nucellar layers die during the seed development [35,39] providing nutrition for the surrounding tissues and later functioning as a barrier against water and fungi [40,41]. The importance of caspase-like VEIDase activity, type II metacaspases, and Tad-D nuclease have been perceived in cell death during Scots pine and Norway spruce (*Picea abies* (L.) Karst.) embryogenesis [35,42,43]. In conifers, the autophagy related (ATG) proteins are essential for vacuolar cell death and for instance, in Norway spruce, depletion of ATG5 and ATG6 causes aberrations in the development of suspensors [44].

Here, we study the progress of two distinct types of cell death programs and reveal the link between PCD and the expression of *CAT* and *RBR* genes which are typically considered as maintainers of cellular homeostasis. We focus on the cell death processes in the ESR and nucellar layers during the Scots pine seed development, and show that *ATG5* previously connected to the regulation of vacuolar cell death also expresses in the cells destroyed via necrotic-like cell death.

## Methods

### Immature and mature Scots pine seeds

One-year-old immature seed cones were collected during the growing period in July from an open-pollinated elite Scots pine (*Pinus sylvestris* L.) clone, K818, growing in the Scots pine clone collection in Punkaharju, Finland (61°48′ N; 29°17′ E). Immature Scots pine seeds were dissected from the developing cones. For the anatomical observations, for the detection of PCD and for the localization of proteins, starch, and mRNA transcripts of *ATG5*, *CAT*, *RBR* and β-glucosidase (*βG*, EC 3.2.1.21), the seed coats were removed and immature seeds were fixed immediately and embedded in paraffin as described below. For the gene expression studies, immature seeds were stored in liquid nitrogen until use.

Mature Scots pine cones were collected from the same grafts as the immature cones in late fall of the same growing season. Mature Scots pine seeds were surface sterilized with 3% Plant Preservative Mixture (Plant Cell Technology) overnight and placed thereafter on moist filter paper and let to imbibe at room temperature (RT) for two days. Thereafter, seeds were opened with a scalpel, seed coats were removed, and the embryos and megagametophytes were separated from each other for the gene expression analysis. For the localization of *ATG5* and *CAT* mRNA transcripts mature seeds were fixed as described below.

### Anatomical and histochemical observations

Developing and imbibed mature Scots pine seeds with and without seed coats were dissected for the histochemical localization of H_2_O_2_ and peroxidase. The 3,5,3′5′-tetramethylbenzidine (TMB) is oxidised by peroxidases in a reaction where H_2_O_2_ acts as a hydrogen acceptor, and thus, TMB can be used to localize H_2_O_2_ and peroxidases [45]. For the H_2_O_2_ localization the seeds were incubated in 50 mM Tris-acetate-buffer (pH 5.0), which contained 0.1 mg/ml 3,5,3′,5′-TMB-HCl [45], at RT at least for 45 min. In addition, seeds were treated with 3% H_2_O_2_ in 1x phosphate-buffered saline (PBS) buffer (10 mM phosphate, 150 mM NaCl, pH 7.4) for 10 min prior to the histochemical staining for the peroxidase location. Negative controls were incubated in 50 mM Tris-acetate-buffer.

The DAB (3,3′-diaminobenzidine) Peroxidase Substrate kit (Vector Laboratories) was used in localizing peroxidase activity following manufacturer’s instructions. The dissected seeds were incubated for two or three min at RT in reaction mixture. The DAB Peroxidase Substrate reaction mixture contains H_2_O_2_ which oxidizes DAB, and subsequently generates a dark brown reaction product when peroxidases are present. Negative controls were incubated in reaction mixture without DAB. The blocking of peroxidase activity was attempted by incubating seed material in 1% H_2_O_2_ at RT for 10 min. The sections were examined by a stereo microscope (Stemi DV4, Carl Zeiss) and imaged with a digital camera (Nikon Coolpix 950, Japan).

Developing and mature Scots pine seeds were fixed for studying the anatomical features of the embryos and for *in situ* mRNA hybridization analyses using the following protocol from fixation to coverslip mounting. Tissues were fixed in 4% (w/v) *p*-formaldehyde in 1x PBS. After dehydration with a graded series of ethanol, ethanol was replaced by tertiary butanol and then gradually by paraffin. Sections (5 and 7 μm) were cut from the embedded samples with a microtome, mounted on SuperFrost®Plus slides (Menzel-Glaser) and fixed by drying overnight at 40°C.

**Figure 1:**
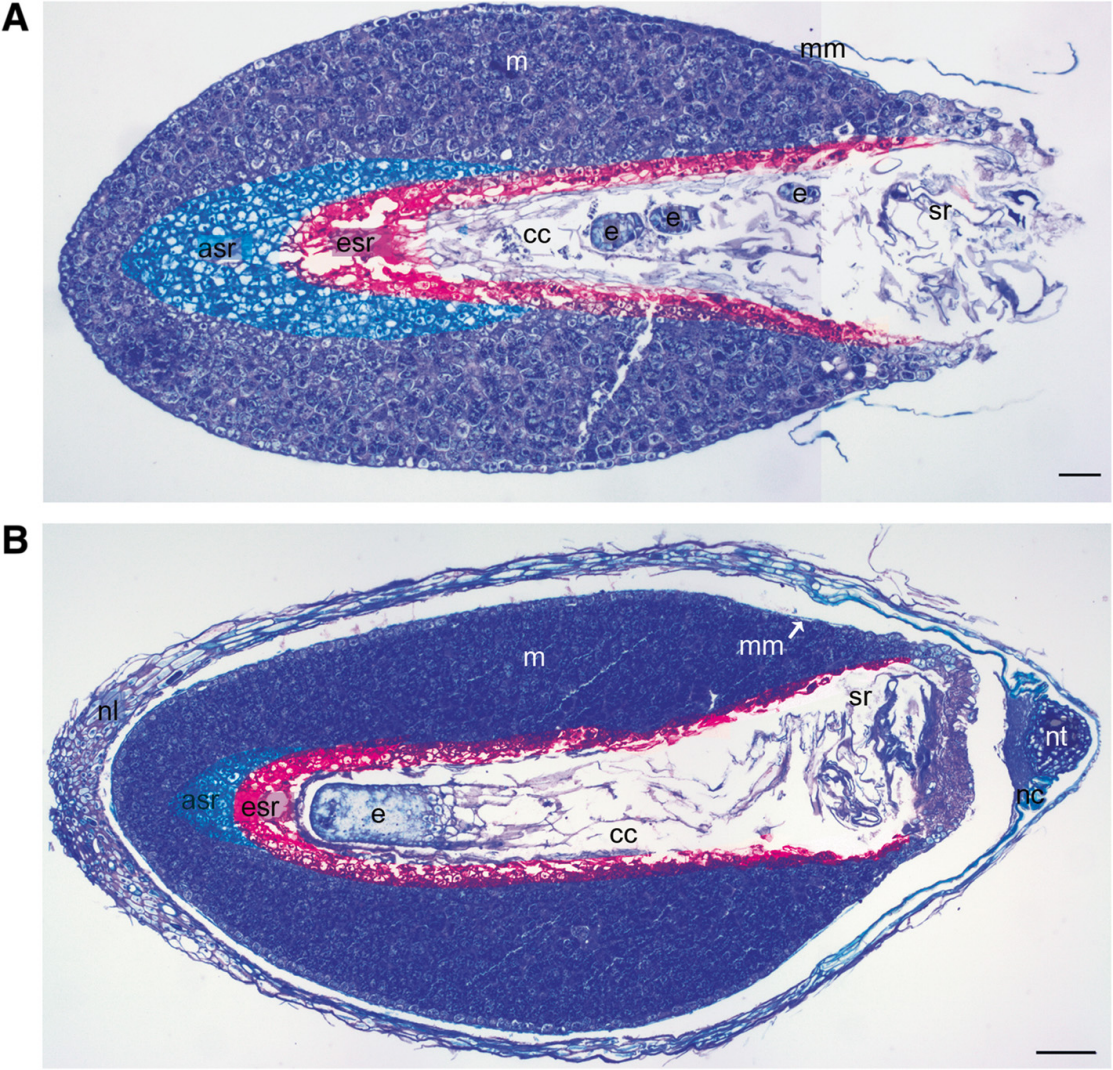
**Early and late developmental stages of Scots pine embryogenesis. (A)** The dominant embryo and subordinate embryos in the corrosion cavity at the early embryogeny surrounded by the embryo surrounding region (esr) of the megagametophyte highlighted with red color. The arrow-shaped region (asr) of the megagametophyte outside the esr highlighted with blue color. **(B)** The dominant embryo in the corrosion cavity at the late embryogeny. The esr of the megagametophyte highlighted with red color and asr highlighted with blue color. asr = arrow-shaped region, cc = corrosion cavity, e = embryo, esr = embryo surrounding region, m = megagametophyte, mm = megaspore membranes, nc = nucellar cap, nl = nucellar layers, nt = cellular nucellus, sr = suspensor remnants. Bars: 100 μm.

The paraffin sections were dewaxed in Histochoice (Sigma) and rehydrated through a graded series of ethanol. To study the developmental stage of the embryos, the preparates were stained with toluidine blue (0.05% toluidine blue in H_2_O). According to Singh [46] Scots pine embryogenesis is divided into three different phases, proembyogeny, early embryogeny and late embryogeny. Here, we studied the early embryogeny which initiates with the elongation of the suspensor system and terminates with the appearance of the root meristem (Figure 1A) and the late embryogeny which contains the establishment of root and shoot meristems and the maturation of the leading embryo (Figure 1B). For the protein and starch visualization the sections were stained with 0.2% amido black [47] and with 0.5% potassium iodide-iodine (IKI) [48], respectively. All sections were studied with a light microscope (Nikon Eclipse E600) and photographed with a Qimaging Micropublisher 5.0 RTV digital camera. Adobe Photoshop CS was used to adjust contrast, brightness and colour uniformly to entire images.

### TUNEL assay

Nuclear DNA fragmentation, i.e. DNA strand breaks leaving free 3′-OH termini, was shown by the TUNEL (terminal deoxyribonucleotidyl transferase (TdT)-mediated deoxyuridine triphosphate (dUTP) nick end labelling) assay. The dewaxed and rehydrated sections were digested with 10 μg/ml proteinase-K (Roche) for 30 min and two washings with PBS. The sections were labelled with TMR red (red fluorescence) using *in situ* cell death detection kit (Roche) according to the manufacturer’s protocol. Prior to the labelling procedure, the positive control sections were incubated with DNase I recombinant (Roche) to induce DNA strand breaks. The label solution without terminal transferase, instead of the TUNEL reaction mixture, was used as a negative control. The labelled sections were examined with a confocal laser scanning microscope (LSM 5 Pascal, Carl Zeiss) with an HBO 100 mercury lamp and using the HeNe laser 543 nm line, dichroic beam splitter (HFT 488/543/633; Carl Zeiss) and LP 560 emission filter (Carl Zeiss).

### RNA extraction, reverse transcription and cDNA cloning

Total RNA was extracted using the automated magnetic-based KingFisher™ mL method (Thermo Electron Corporation) with the MagExtractor® total RNA purification kit (Toyobo) according to the manufacturer’s instructions. The RNA samples were treated with RNase-free DNase (Invitrogen) at RT for 15 min for the elimination of contaminating genomic DNA. Thereafter the RNA samples were purified with the NucleoSpin® RNA Clean-Up kit (Macherey-Nagel). The RNA yields were measured three times with OD_260_ analysis using NanoDrop ND1000 spectrophotometer (NanoDrop Technologies), and 1 μg of each RNA sample was subsequently used for the cDNA synthesis. cDNA was reverse-transcribed from an anchored oligo-dT primer by SuperScript II reverse transcriptase (Invitrogen) using standard methods in a reaction volume of 20 μl. Fragments of the putative Scots pine *ATG5* and cell wall associated *βG* were amplified by standard PCR using cDNA from immature seeds as a template, gene specific primers (Additional file 1: Table S1) and DyNAzyme™EXT polymerase (Finnzymes). The fragments with appropriate length were gel-purified by Montage DNA Gel Extraction Kit (Millipore Corporation), cloned by TOPO TA Cloning Kit (Invitrogen) and sequenced by an Applied Biosystems 3730 DNA analyzer.

### *In situ* mRNA hybridization analysis

The RNA probes for the *in situ* mRNA hybridization analyses of *ATG5*, *CAT*, *RBR* and *βG* transcripts in developing and mature seeds were prepared using a PCR-based technique [49]. The T7 RNA polymerase promoter sequence (TAATACGACTCACTATAGGG) was introduced at the 5′ends of the gene-specific primers (Additional file 1: Table S2). The downstream primers contained an artificially introduced T7 promoter at its 5′end, which enabled the synthesis of antisense transcripts. The upstream primers containing T7 promoters at the 5′ends was used for the synthesis of sense transcripts, i.e. as negative control.

The PCR fragment representing the coding region of either *ATG5*, *CAT*, *RBR* or *βG* was produced under standard PCR conditions by DyNAzyme™EXT polymerase (Finnzymes) using plasmid DNA containing the cDNA in question as a template. The PCR fragment was gel-purified (DNA Gel Extraction Kit, Millipore Corporation) and 250 ng was subsequently used as a template DNA for *in vitro* transcription by T7 RNA polymerase (Invitrogen), incorporating dig-UTP via DIG RNA labelling Mix (Roche Molecular Biochemicals). Template DNA was digested with four units of RNase-free DNase (Invitrogen) in a reaction volume of 20 μl at 37°C for 10 min, the probe was precipitated and the yield of the DIG-labeled RNA probe was estimated by comparing the intensity of the sample to the defined controls made with DIG-labelled control RNA (Roche Molecular Biochemicals).

The sections were dewaxed, treated with Proteinase K (Finnzymes, 1 μg/ml in 100 mM Tris/HCl, 50 mM Na_2_EDTA, pH 7.5) at 37°C for 30 min and dehydrated in a graded series of ethanol up to absolute. The slides were dried in a vacuum for 1 h. For the hybridization, 250 μl of hybridization mixture was added to the sections, mounted under coverslips and incubated in a water atmosphere at 55°C overnight. The hybridization mixture contained 0.8 μg/ml of DIG-labelled RNA antisense or sense probe, 50% (v/v) deionized formamide, 300 mM NaCl, 10 mM Tris/HCl (pH 7.0), 10 mM Na_3_PO_4_ (pH 7.0), 50 mM EDTA, 10% (w/v) dextran sulfate, 200 μg/ml tRNA, 1x Denhardt’s solution and 10 units/ml RNase OutTM inhibitor (Invitrogen).

After the hybridization, the slides were washed in 2x NaCl/Cit (300 mM NaCl, 30 mM sodium citrate, pH 7.0) at RT for 10 min followed by 1x and 0.5x NaCl/Cit treatment by slow shaking at 37°C for 10 min. The washing was followed by treatment of the RNase A (Roche, 1 μg/ml in 10 mM Tris/HCl, 500 mM NaCl, 1 mM Na_2_EDTA, pH 7.5) at 37°C for 60 min. Then the slides were washed four times in the same solution but without RNase A in a slow shaking at 37°C for 15 min and once in 2x NaCl/Cit at RT for 30 min.

For the detection of hybridized probe, the slides were washed in Tris/NaCl buffer (100 mM Tris/HCl, 150 mM NaCl, pH 7.5) for 5 min and blocked with 3% (w/v) blocking reagent (Dig Nucleic acid detection Kit, Roche) and 0.3% (v/v) Triton X-100 in Tris/NaCl buffer in a water atmosphere at RT for 30 min. 1 unit/ml of Anti-DIG-AP Fab fragments (Roche) in Tris/NaCl buffer was added to the sections, mounted under parafilm and incubated in a water atmosphere at RT for 2 h. The slides were washed at RT four times in Tris/NaCl buffer for 10 min and in AP buffer (100 mM Tris/HCl, 100 mM NaCl, 50 mM MgCl_2_, pH 9.5) for 5 min. For colour development, 2% (v/v) NBT/BCIP substrate (Dig Nucleic acid detection Kit, Roche) and 1% (v/v) Triton X-100 in AP buffer was dispersed on the sections, mounted with coverslips and incubated in a water atmosphere at RT in the dark overnight. The slides were washed with water, dehydrated in a graded series of ethanol, air-dried and then mounted with immersion oil and covered with coverslips. The sections were examined with a light microscope (Nikon Optiphot 2, Japan) and imaged with an Infinity*1*–*3*C camera (Lumenera Corporatiom, Ottawa, Ontario, Canada), using the IMT iSolution Lite image-processing program (IMT i-Solution Inc., Vancouver, BC, Canada). The auto and manual tiling feature of image-processing program was used to combine separate images to one. Non-specific signal was observed in the remnants of the degenerated suspensors, ESR, and inner nucellar layers generated by fragmented nucleic acids as previously described in Vuosku *et al*. [50]. Adobe Photoshop CS5 was used to adjust contrast, brightness and colour uniformly to entire images.

### Quantification of gene expression

The real-time reverse-transcription PCR analysis (Q-RT-PCR) was used for the quantification of the *CAT*, *RBR* and *βG* expression in immature seeds at the developmental stages of early and late embryogeny as well as in the embryos and megagametophytes of mature seeds. Being aware of the challenges in finding suitable endogenous reference genes for pine embryogenesis [51], both absolute and relative Q-RT-PCR analyses were used. For the determination of mRNA copy numbers in the absolute quantification, the standard curves were generated using serial 10-fold dilutions of synthesized RNA molecules to control variability during both RT and PCR steps of Q-RT-PCR runs. The DNA templates from which the RNA molecules could be transcribed were amplified by basic PCR procedure using gene-specific primers (Additional file 1: Table S3). The upstream primers contained T7 promoter sequence (TAATACGACTCACTATAGGG) and the downstream primers contained poly(T) tail at their 5′end. The DNA molecules were subsequently used as templates for *in vitro* transcription by T7 RNA polymerase. The numbers of standard RNA molecules added to the reverse-transcription reactions were calculated using the molecular weights of the oligonucleotides and Avogadro’s constant (6.022 · 10^23^ mol^−1^). In the relative quantification, ubiquinone (*UBI*) and glyceraldehyde-3-phosphate dehydrogenase (*GAPDH*) were used as endogenous reference genes (see Additional file 2 for details).

In the absolute Q-RT-PCR analysis (see Additional file 2 for details for the relative quantification), the number of biological replicates was 5, 5, 6, and 4 for early stage immature seeds, late stage immature seeds, embryos, and megagametophytes, respectively. The PCR amplification conditions of the gene fragment were optimized for the LightCycler® 2.0 instrument (Roche Diagnostics), and the subsequent PCR runs showed a single PCR product during melting curve (The Tm Calling Analysis of Lightcycler® 480 Software release 1.5.0 SP3) and electrophoretic analysis. The real-time PCR amplifications were performed using the LightCycler® 480 SYBR Green I Master (Roche), 50 nM gene specific primers (Additional file 1: Table S4) and 2 μl cDNA in the reaction volume of 20 μl. The negative controls contained 2 μl of molecular grade water instead of cDNA template. The real-time PCR amplification was initiated by incubation at 95°C for 10 min followed by 45 cycles: 10 s at 95°C, 10 s at 58°C and 5 s at 72°C. Every PCR reaction was done as duplicate to control for the variability of PCR amplification and the coefficient variation, CV%, of technical replicates was < 2.5. The Abs Quant/2nd Derivative analysis of Lightcycler® 480 software release 1.5.0 SP3 was utilized to generate the crossing point and concentration values for each sample.

### Statistical analysis

The mRNA copy numbers generated with the absolute Q-RT-PCR analysis were utilized in calculating the gene expression. The expression of studied genes was calculated by dividing the individual values with the mean number of transcripts in the early embryogeny, that is, value of one was given to the gene expression in the early embryogeny. The significance of difference between the early and late embryogeny and between the embryo and megagametophyte of mature seeds was examined using two sample *t* test with the graphical user interface R Commander [52] in R software package (2.11.0) [53]. Log 10 transformation was conducted to variables not normally distributed.

## Results

### Nuclear DNA fragmentation in ESR and nucellar layers

In our previous study, we showed that megagametophyte cells in the ESR are destroyed by morphologically necrotic cell death and also the cells in the megagametophyte surrounding nucellar layers die during the Scots pine seed development [35]. Likewise, TUNEL positive nuclei were detected in the ESR cells and in the nucellar layers in this study (Additional file 3: Figure S2).

### Transient decrease of *CAT* expression in megagametophyte at late embryogeny

The Scots pine *CAT* (EU513163) was amplified using zygotic embryo cDNA as a template. Previously, three *CAT* genes have been found present in the angiosperm species tobacco (*Nicotiana tabacum* L.), Arabidopsis, maize, pumpkin (*Cucurbita* sp.) and rice (*Oryza sativa* L.) [54-58]. The angiosperm class I CATs are strongly expressed in photosynthetic tissues, whereas class II CATs are associated with vascular tissues. Class III CATs are expressed especially in seeds and reproductive tissues [25]. Scots pine *CAT* showed high sequence similarity (75%) with maize *CAT1* belonging to class III.

**Figure 2:**
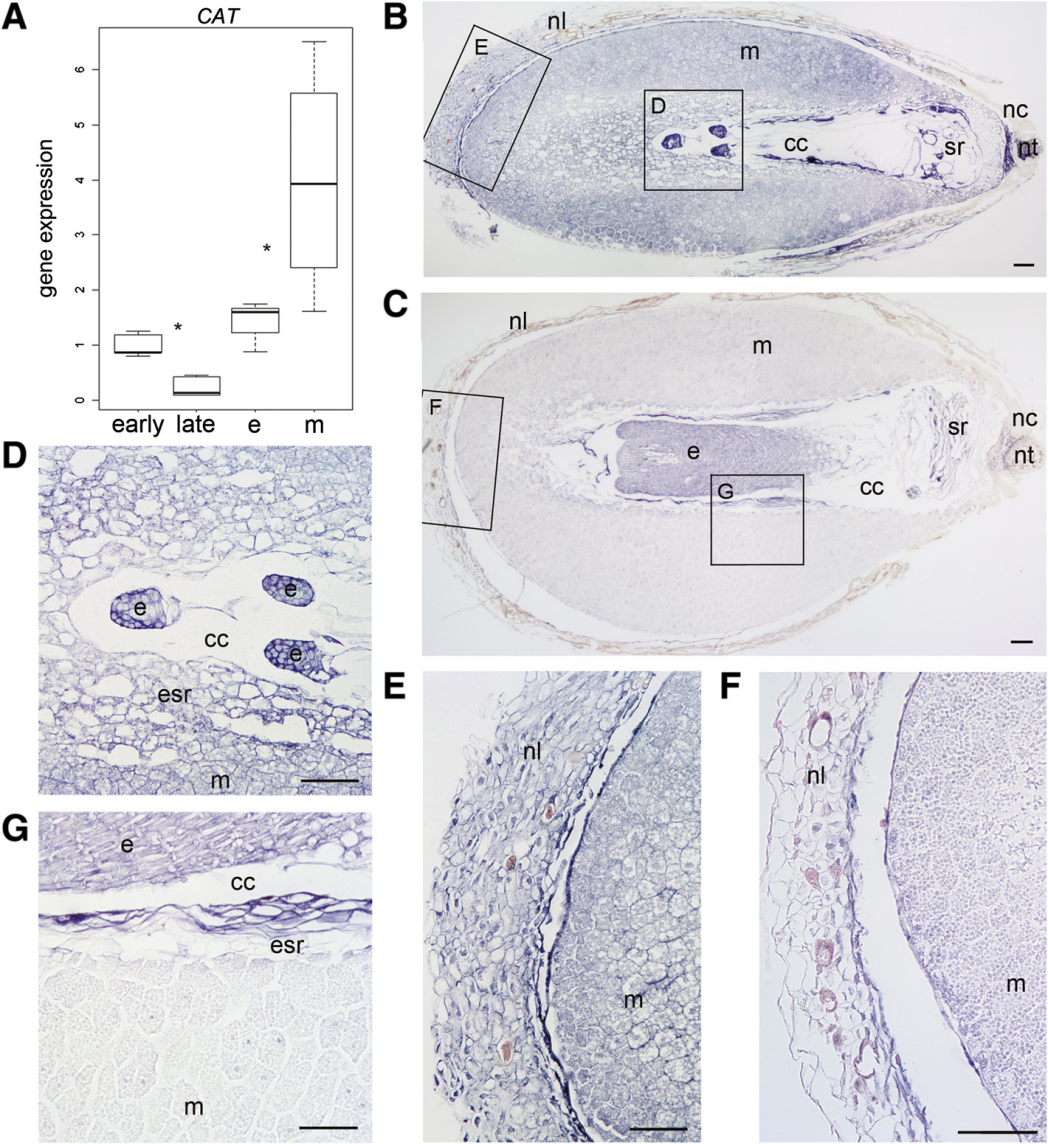
***CAT*** **expression in developing and mature Scots pine seeds. (A)** The relative expression of *CAT* in developing seeds at the early and late embryogeny and in the embryos (e) and megagametophytes (m) of mature seeds. The expression was based on mRNA copy numbers generated with the absolute Q-RT-PCR analysis and values presented were normalized using the expression at the early embryogeny. A star denotes significant (*P* <0.05) difference in the expression. **(B)** The localization of *CAT* mRNAs by *in situ* hybridization with DIG-labelled RNA-probes (blue signal) in a developing Scots pine seed at the early embryogeny. **(C)** The localization of *CAT* mRNAs at the late embryogeny. **(D)** Intense *CAT* expression in the developing embryos and in the megagametophyte at the early embryogeny. **(E)** Intense *CAT* expression in the nucellar layers at the early embryogeny. **(F)** Minor *CAT* expression in the nucellar layers at the late embryogeny. **(G)** Intense *CAT* expression in the cells of the leading embryo and minor *CAT* expression in the megagametophyte cells at the late embryogeny. cc = corrosion cavity, e = embryo, esr = embryo surrounding region, m = megagametophyte, nc = nucellar cap, nl = nucellar layers, nt = cellular nucellus, sr = suspensor remnants. Bars: 100 μm.

**Figure 3:**
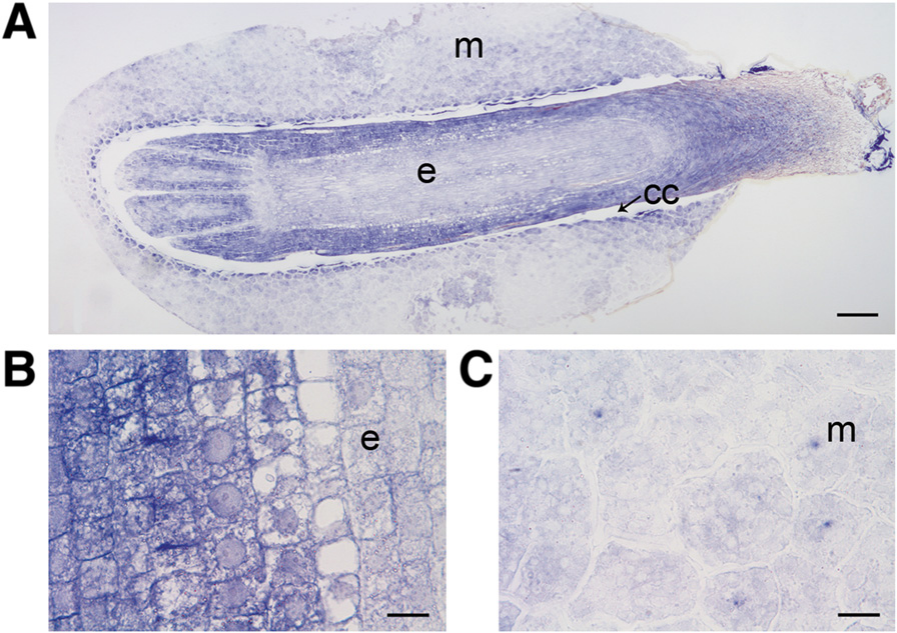
**The localization of** ***CAT*** **mRNAs in a mature Scots pine seed. (A)** Intense *CAT* expression (blue signal) in the embryo and in the megagametophyte cells surrounding the corrosion cavity. **(B)** Intense *CAT* expression in the embryonic cells. **(C)** Minor *CAT* expression in the cells in the inner part of the megagametophyte. cc = corrosion cavity, e = embryo, m = megagametophyte. Bars: **(B, C)** 20 μm and **(A)** 200 μm.

Based on the Q-RT-PCR analysis, the number of *CAT* mRNA transcripts decreased significantly in developing Scots pine seeds when the embryogenesis proceeded from the early to the late stage (*P* <0.001). In mature seeds, the number of *CAT* transcripts was significantly greater in megagametophytes than in embryos (*P* =0.02) (Figure 2A). At the early embryogeny, intense *CAT* expression was localized in the cells of both the dominant embryo and subordinate embryos (Figure 2B and D) as well as in the cells of the megagametophyte and the nucellar layers (Figure 2B and E). At the late embryogeny, the *CAT* expression was still strong in the cells of the dominant embryo (Figure 2C and G), whereas it was clearly diminished in the cells of the nucellar layers (Figure 2C and F) and in the megagametophyte (Figure 2C and G). In mature seeds the megagametophyte cells surrounding the corrosion cavity showed intense *CAT* expression whereas in the embryo, the expression was comparable to the late embryogeny (Figure 3). The specificity of the antisense *CAT* probe was confirmed by the absence of signals in the sections hybridized with the sense *CAT* probe (Additional file 4: Figure S3).

### Peroxidase and H_2_O_2_ localization

**Figure 4:**
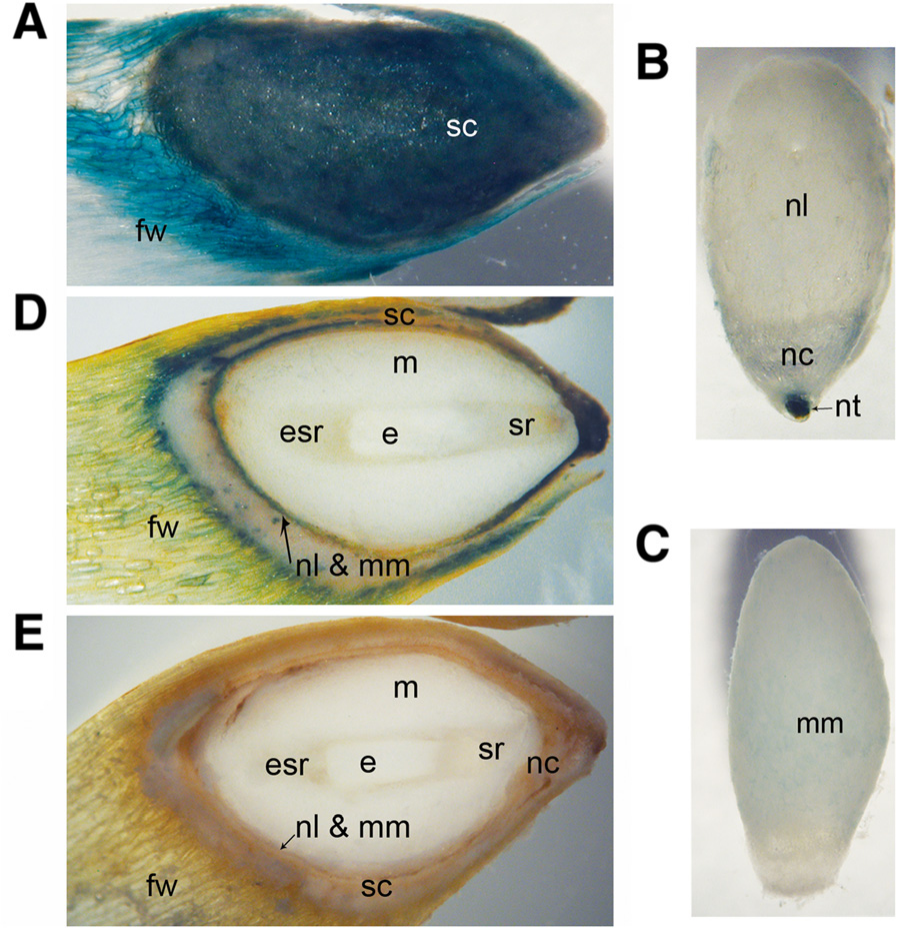
**The localization of H**_**2**_ **O**_**2**_ **in developing Scots pine seeds. (A)** Intense blue colour from TMB indicates the presence of H_2_O_2_ in the seed coat and flight wing at the early embryogeny. **(B)** The localization of H_2_O_2_ in the nucellar cap, cellular nucellus, and in the nucellar layers at the early embryogeny. **(C)** The localization of H_2_O_2_ in the megaspore membranes at the early embryogeny. **(D)** The minor amount of H_2_O_2_ in the seed coat and flight wing and the large amount of H_2_O_2_ indicated by the dark blue colour in the nucellar layers at the late embryogeny. **(E)** The seed treated with Tris-acetate buffer without TMB. cc = corrosion cavity, e = embryo, esr = embryo surrounding region, fw = flight wing, m = megagametophyte, mm = megaspore membranes, nc = nucellar cap, nl = nucellar layers, nt = cellular nucellus, sc = seed coat, sr = suspensor remnants.

**Figure 5:**
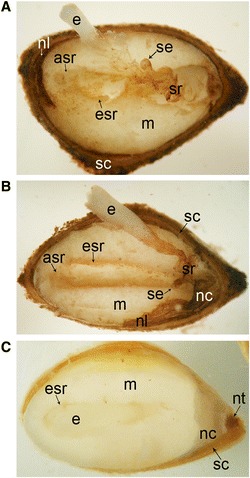
**The localization of peroxidase activity in developing Scots pine seeds. (A**, **B)** Oxidized DAB (brown colour) indicated peroxidase activity in the seed coat, nucellar layers, megagametophyte cells surrounding the corrosion cavity, suspensor cells and subordinate embryos at the early **(A)** and late **(B)** embryogeny. **(C)** The seed treated with the reaction buffer without DAB. asr = arrow-shaped region, e = embryo, esr = embryo surrounding region, m = megagametophyte, mm = megaspore membranes, nc = nucellar cap, nl = nucellar layers, nt = cellular nucellus, sc = seed coat, se = subordinate embryo, sr = suspensor remnants.

At the early embryogeny, intense blue colour indicating H_2_O_2_ presence was observed in the seed coat, flight wing (Figure 4A), and cellular nucellus (Figure 4B). In addition, H_2_O_2_ was detected in the nucellar layers (Figure 4B), and megaspore membranes (Figure 4C). At the late embryogeny, H_2_O_2_ was still present in the seed coat and flight wing, but the extent was clearly diminished (Figure 4D). The seeds treated with Tris-acetate buffer were used as negative control (Figure 4E). Peroxidase activity was detected throughout the embryogenesis in the very same seed structures as H_2_O_2_ and, additionally, in the ESR cells and in the arrow-shaped region (ASR) of megagametophyte, suspensor cells, and subordinate embryos (Figure 5A and B, Additional file 5: Figure S4). For negative control DAB was excluded from the reaction buffer (Figure 5C). The peroxidase localization with DAB and TMB protocol supplemented with H_2_O_2_ also gave comparable results (Additional file 5: Figure S4).

### Minute *RBR* expression in dying megagametophyte cells in ESR

**Figure 6:**
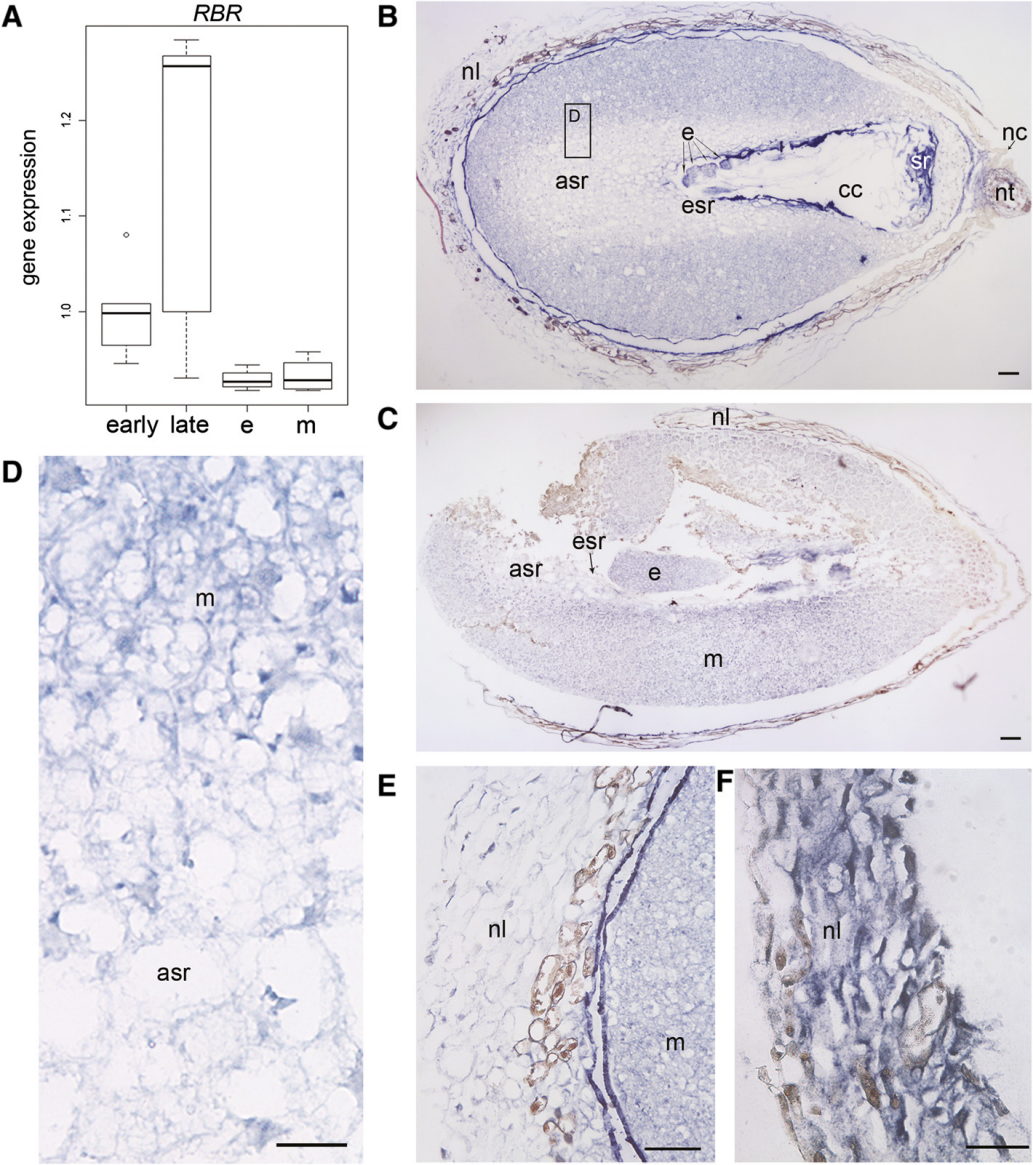
***RBR*** **expression in developing Scots pine seeds. (A)** The relative expression of *RBR* in developing seeds at the early and late embryogeny and in the embryos (e) and megagametophytes (m) of mature seeds. The expression was based on mRNA copy numbers generated with the absolute Q-RT-PCR analysis and values presented were normalized using the expression at the early embryogeny. **(B)** The localization of *RBR* mRNAs (blue signal) in a developing Scots pine seed at the early embryogeny. **(C)** The localization of *RBR* mRNAs at the late embryogeny. **(D)** Minor *RBR* expression in the ESR of the megagametophyte at the early embryogeny. **(E)** Weak *RBR* expression in the cells of the nucellar layers at the early embryogeny. **(F)** Intense *RBR* expression in the cells of the nucellar layers at the late embryogeny. asr = arrow-shaped region, cc = corrosion cavity, e = embryo, esr = embryo surrounding region, m = megagametophyte, nc = nucellar cap, nl = nucellar layers, nt = cellular nucellus, sr = suspensor remnants. Bars: **(D)** 20 μm, **(F)** 50 μm, and **(B, C, E)** 100 μm.

The predicted Scots pine RBR protein showed 57% identity with Arabidopsis RBR 1 protein. In Arabidopsis, only one *RBR* gene has been found, and the presence of two distinct *RBR* genes have been suggested to a unique feature of grasses [59]. In developing Scots pine seeds, there was overall low variation in the number of *RBR* mRNA transcripts across the studied Scots pine seed material. The *RBR* transcript levels were comparable between early and late embryogenesis as well as between the embryos and megagametophytes of mature seeds when the expression was quantified with Q-RT-PCR (Figure 6A). The *RBR* expression was localized in both embryonic and megagametophyte cells throughout the embryogenesis excluding the dying megagametophyte cells in the ESR and the ASR cells (Figure 6B, C and D, Additional file 6: Figure S5). The *RBR* expression signal was found weak in nucellar cells at early embryogeny, but showed more intensity at later stages of embryogenesis (Figure 6E and F). The specificity of the antisense *RBR* probe was confirmed by the absence of signals in the sections hybridized with the sense *RBR* probe (Additional file 4: Figure S6).

### *ATG5* expression in dying cells of ESR and nucellar layers

**Figure 7:**
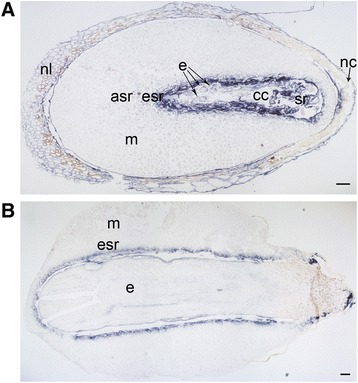
**The localization of** ***ATG5*** **expression in developing and mature Scots pine seeds. (A)** *ATG5* expression (blue signal) in the esr of the megagametophyte and in the nucellar layers at the early embryogeny. **(B)** *ATG5* expression in the esr of the megagametophyte and in the maturating tracheids of the embryo in a mature seed. asr = arrow-shaped region, cc = corrosion cavity, e = embryo, esr = embryo surrounding region, m = megagametophyte, nc = nucellar cap, nl = nucellar layers, sr = suspensor remnants. Bars: 100 μm.

In order to study the type of the cell death processes in the ESR of the megagametophyte and in the nucellar layers, we sequenced the complete coding sequence of the Scots pine *ATG5* gene (KM046993) and localized *ATG5* mRNA transcripts in developing Scots pine seeds at the early embryogeny and in mature seeds after two days of imbibition. The predicted Scots pine ATG5 protein showed 95% identity with the Norway spruce ATG5 protein which has previously shown to be essential for autophagy and vacuolar cell death [44]. At the early embryogeny, the *ATG5* expression was localized in both the ESR of the megagametophyte and the nucellar layers (Figure 7A). In a mature seed, *ATG5* expressed still in the ESR, but also in the maturating tracheids (Figure 7B). The specificity of the antisense *ATG5* probe was confirmed by the absence of signals in the sections hybridized with the sense *ATG5* probe (Additional file 4: Figure S7).

### Accumulation and release of starch and storage proteins during Scots pine seed development

**Figure 8:**
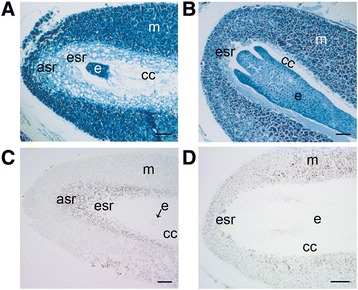
**Histochemical localization of proteins by amido black and starch by potassium iodide**-**iodine in developing Scots pine seeds. (A)** Megagametophyte cells with few protein storage vacuoles at the early embryogeny. **(B)** Megagametophyte cells stocked with protein storage vacuoles at the late embryogeny. **(C)** Megagametophyte cells with starch grains in the vicinity of the corrosion cavity at the early embryogeny. **(D)** Starch grains in the cotyledons and root meristem of a late embryo as well as in the megagametophyte. asr = arrow-shaped region, cc = corrosion cavity, e = embryo, esr = embryo surrounding region, m = megagametophyte. Bars: **(A, C)** 100 μm and **(B, D)** 200 μm.

The Scots pine seed development comprises the accumulation of storage compounds such as starch and storage proteins. At the early embryogeny, the megagametophyte cells had only few storage proteins (Figure 8A) but during the late embryogenesis intensively stained protein bodies were found in the megagametophyte tissue (Figure 8B). However, the megagametophyte cells in ESR in addition to the cells in ASR did not pile up proteins during early embryogeny, but instead contained high quantity of starch (Figure 8C). As the embryo development proceeded the cell wall and plasma membrane of megagametophyte cells in ESR were destroyed with the release of starch grains into the corrosion cavity. In dying cells, the cell wall weakening and breakdown seemed to be connected with the increased intensity of the *βG* gene expression detected with *in situ* hybridization (Additional file 7: Figure S8, S9). In addition to the ESR and ASR, starch also accumulated into the cells of both the developing embryo and megagametophyte (Figure 8D).

## Discussion

As an orthodox seed, a developing Scots pine seed experiences three characteristic features, embryo development, accumulation of reserve compounds and maturation/drying, which lead from a zygotic embryo to a mature, quiescent seed [60]. Both metabolic activity and moisture content fluctuate drastically during seed development [61-64]. Thus, the sources of ROS production, connected to basic cellular and specific seed developmental processes, in cells also vary [65]. During the seed development, the cell cycle is tightly controlled for proper growth and development [66] but also for cellular level oxidative stress responses [67]. Here, we show the tissue and developmental stage specific expression of the *ATG5*, *CAT*, and *RBR* genes as well as the connection between the gene expressions and cell death programs during the Scots pine seed development.

The development of a viable Scots pine seed includes the strictly coordinated action of several cell death programs (reviewed by Vuosku *et al*. [68]). The autophagic PCD of subordinate embryos as well as their suspensor tissues is initiated already at early embryogeny [34]. The nucellar layers face destruction at late embryogeny [35] to be used as a nutrition for the surrounding tissues of the seed and later on to form an efficient barrier to the passage of water and against fungal infections [40,41]. The necrotic-like death of the megagametophyte cells in the ESR continues throughout the embryogenesis. In the present study, the ESR cells differed already at the early embryogeny from the other megagametophyte cells in the expression of *ATG5*, *CAT*, *RBR* and *βG* genes as well as the storage compound distribution. The ESR cells had higher amount of starch and less storage proteins than the surrounding cells of the megagametophyte. The starch accumulation has been shown to precede PCD of the endosperm in several cereals [69-72] and in the perisperm of quinoa (*Chenopodium quinoa* Willd.) [73]. The role of starch accumulation may be more general in PCD: the endosperm cells of starch-deficient maize mutants contained more ethylene and faced accelerated PCD [69]. In cereals, the storage compounds are not evenly located in endosperm, but there is a gradient in the distribution of storage proteins and starch [69,74]. In a Scots pine seed, protein bodies and starch, however, filled all megagametophyte cells except the cells in the ESR at the late embryogeny.

Intense *βG* expression was found in the ESR of the megagametophyte and in the nucellar layers which suggests that during the cell death processes *β*G contributes to either weakening or breaking down of cell walls depending on the type and physiological function of the cell death. In Arabidopsis, cell wall associated *βG* breaks down polysaccharides to soluble sugars, and is induced by both starvation [75] and senescence [76]. In barley (*Hordeum vulgare* L.), hydrolysis of *β*-linked oligosaccharides results in cell wall degradation in endosperm during seed germination [77]. Hence, *β*G appears to contribute to both the degradation of oligosaccharides generated in cell wall turnover and release of monolignols from their glycosides for the stabilization of secondary cell wall by lignification [78].

Generally, CATs are considered as a sink for H_2_O_2_ [25,79]. In the present study, the tissue and developmental stage specific *CAT* expression suggested an important role for CAT in enzymatic ROS protection in Scots pine seeds. *CAT* showed intense expression in megagametophyte cells at the early embryogeny simultaneously with the accumulation of starch and storage proteins. Interestingly, the *CAT* expression signal faded out in the megagametophyte when the embryogenesis proceeded to the late stage but increased again during the imbibition phase of the seed germination. The transient decrease in the *CAT* expression co-occurred with slight DNA fragmentation in the nuclei of megagametophyte cells – that is to say, an incipient sign of cell death – detected in our previous study [35]. Unlike in the megagametophyte, *CAT* expression was intense in the cells of developing embryos throughout the embryogenesis and also in embryonic cells of mature seeds. Previously, CAT protein has been purified from the megagametophyte of loblolly pine (*Pinus taeda* L.) seeds [80], and the *CAT* gene has also been found to be active in *in vitro* cultured Scots pine embryogenic cells [31]. It has been suggested that in white spruce (*Picea glauca* (Moench.) Voss) the interplay of CAT and the ubiquitin mediated proteolytic system regulates ROS production and subsequent cell death in the megagametophyte during germination [81].

The H_2_O_2_ localization overlapped with the *ATG5* and *CAT* localizations at early embryogeny in the nucellar layers which undergo PCD later during Scots pine seed development [35,39]. Together the *CAT* expression and the presence of peroxidases in several seed structures indicate active H_2_O_2_ scavenging, and moreover, strict regulation of H_2_O_2_ levels during the embryogenesis. Possibly, the H_2_O_2_ detected in the nucellar layers exceeded the detoxifying capacity of ROS detoxifying enzymes, including CAT, thus triggering PCD, the mechanism also proposed by He and Kermode [81] for ROS mediated death of white spruce megagametophyte. Additionally, H_2_O_2_ can be involved in growth and developmental processes due to its role as an important signalling molecule [82]. Previously, high CAT activity has been detected in durum wheat (*Triticum durum* Desf.) kernels before the start of maturation drying [83], and CAT has also found to affect seed desiccation of sunflower (*Helianthus annuus* L.) by preventing dehydration-related oxidative damage [84]. Here, the reduced *CAT* expression in the megagametophyte cells at the late embryogeny, however, suggests that in Scots pine seeds the CAT protection against H_2_O_2_ damage was more connected to active metabolism than to dehydration-related oxidative stress during the seed desiccation.

We found that *RBR* expression remain quite stable throughout the Scots pine embryogenesis as well as in both the embryos and megagametophytes of mature seeds. In Arabidopsis, the loss of RBR function is gametophyte-lethal because the mature unfertilized mutant megagametophyte fails to arrest mitosis and undergoes excessive nuclear proliferation in the embryo sac [85]. In the maize endosperm, the down-regulation of *RBR1* by RNAi enhanced cell death and stimulated both the mitotic and endoreduplication cell cycles. Furthermore, DNA content was increased 43% by the down regulation of RBR1 during endosperm development, whereas storage protein content and kernel weight were essentially not affected [15]. In endoreduplication, a cell replicates chromosomal DNA without an intervening mitosis resulting in a higher ploidy level [86]. *Pinus* sp. exhibits the uniform haploid megagametophyte compared to the diploid DNA content of the corresponding embryo [87-89] indicating that no endoreduplication occurs in the megagametophyte cells. In the present study, the minor *RBR* expression found in the ESR suggests that *RBR* down regulation precedes cell death in Scots pine megagametophyte cells. The dying cells of the nucellar layers, however, showed intense *RBR* expression at the late embryogeny. The opposite *RBR* expression goes together with the different morphological features and different magnitudes of cell corpse processing in the ESR of the megagametophyte and in the nucellar layers emphasizing the specific developmental tasks of the cell death processes. Unlike in the ESR, cell death in the nucellar layers may be connected to oxidative stress leading to cell cycle arrest and preventing the replication of damaged DNA by intense *RBR* expression. Further protein level studies are, however, needed to confirm the possible role of RBR in the cell death processes during the Scots pine seed development. The stable *RBR* expressions detected in the present study is concordant with the previous findings revealing that in mammalian cells the pRB protein exhibits only slight variation at the gene expression level but significant differences in the protein phosphorylation status [90].

## Conclusions

Starch accumulation precedes necrotic-like PCD in the ESR cells of the Scots pine megagametophyte, a phenomenon detected previously in the endosperm and perisperm of angiosperm seeds. Furthermore, the changes in the *RBR*, *ATG5* and *βG* expressions specifically in the cells doomed to death via morphologically necrotic cell death in the ESR and ASR of the megagametophyte suggest the genetic regulation of developmental necrosis. The results suggest that the function of CAT in the megagametophyte could be connected to the ongoing metabolism, whereas the continuous *CAT* expression in the embryo underlines the importance of CAT in the preventing of the transfer of H_2_O_2_ damaged components, especially DNA, to the next generation.

## Additional files

Additional file 1:
**PCR primers for** ***in situ*** **mRNA hybridization assays and Q-RT-PCR.**
Additional file 2:
**The determination of gene expression using relative quantification.**
Additional file 3:
**Detection of nuclear DNA fragmentation by TUNEL assay.**
Additional file 4
***In situ*** **mRNA hybridizations with sense probes.**
Additional file 5:
**Verification of DAB visualized peroxidase location with H**_**2**_ **O**_**2**_ **supplemented TMB.**
Additional file 6:
***RBR*** **localization with** ***in situ*** **mRNA hybridization in the transition from early to late developmental stage of the Scots pine seed development.**
Additional file 7:
***βG*** **expression in developing Scots pine seeds.**

## Abbreviations

ATG: Autophagy
*β*G: *β*-glucosidase
CAT: Catalase
ESR: Embryo surrounding region
PCD: Programmed cell death
RBR: Retinoblastoma related protein
ROS: Reactive oxygen species
