# Supplementary material for: Expression of catalase and retinoblastoma-related protein genes associates with cell death processes in Scots pine zygotic embryogenesis
Source: BMC Plant Biol. 2015 Mar 15;15:88. doi: 10.1186/s12870-015-0462-0 (PMC4396594; doi:10.1186/s12870-015-0462-0)
Supplement: Additional file 1: — PCR primers for in situ mRNA hybridization assays and Q-RT-PCR. [file 12870_2015_462_MOESM1_ESM.pdf]

Additional file 1

Table S1. The PCR primers for the amplification of the cDNA fragments of the Scots pine *ATG5* and *βG* genes.

Gene	Sequence of the upstream PCR primer (5′-> 3′)	Sequence of the downstream PCR primer (5′-> 3′)	PCR product size
ATG5	ATGGAGGAGGCAATGCAAGA	TGCCGTAAATACAAGCGAAG	769
βG	CGCTCCGTTTTAGCAGACAT	ATGTGTGCGCTTTGATTAGC	1596

Table S2. The PCR primers for the amplification of the probes for the mRNA *in situ* hybridization assays of the *ATG5***,** *βG, CAT* and *RBR* transcripts.

Gene	Sequence of the upstream PCR primer (5′-> 3′)	Sequence of the downstream PCR primer (5′-> 3′)	PCR product size
ATG5	GGGTGAAGACAGCGTGAAAT	CAGAAGCACCATCCTCAACA	209
βG	CGTGACATTGTTCCATTTCG	GACGAGCAATTCCCTGTCAT	258
CAT	AACCACAGTCATGCAACCAA	AGACCAGGACCAAATGCAAG	245
RBR	CCGAATGTTGATACCCCATC	TGCACAAGTCTCACCACCTC	225

Table S3. PCR primers for the PCR amplification of the standard molecules for the quantitative real-time PCR of the Scots pine *βG, CAT* and *RBR* genes.

Gene	Sequence of the upstream PCR primer (5′-> 3′)	Sequence of the downstream PCR primer (5′-> 3′)	PCR product size
βG	CGTGACATTGTTCCATTTCG	GATCCAAAAACCATCGGAGA	464
CAT	TTTGTTCGCGATGGTATGAA	GACCATGCGTCCTACAGGTT	492
RBR	CCTACACGGCCAAATCCTTA	CATCAGACTCACTGGGAGCA	464

Table S4. PCR primers for the quantitative real-time PCR of the Scots pine *βG, CAT* and *RBR* genes.

Gene	Sequence of the upstream PCR primer (5′-> 3′)	Sequence of the downstream PCR primer (5′-> 3′)	PCR product size
βG	CAAATCTGTTTGTGCCGTTG	CTGACGAGCAATTCCCTGTT	105

CAT	GGGAGGCAAACCTATGTGAA	TTGGTTGCATGACTGTGGTT	110
RBR	ACAGGAAGCAACCTCAGTGC	TCCACTGTCTCATGCCCTAA	92
