# Supplementary material for: Expression of catalase and retinoblastoma-related protein genes associates with cell death processes in Scots pine zygotic embryogenesis
Source: BMC Plant Biol. 2015 Mar 15;15:88. doi: 10.1186/s12870-015-0462-0 (PMC4396594; doi:10.1186/s12870-015-0462-0)
Supplement: Additional file 2: — The determination of gene expression using relative quantification. [file 12870_2015_462_MOESM2_ESM.pdf]

Additional file 2

To validate the expression generated using absolute quantification utilizing synthesized RNA molecules representing the target genes (*CAT*, *RBR*, and *βG*), the relative expression was calculated using two reference genes. The primers for the reference genes, glyceraldehyde-3-phosphate dehydrogenase (*GAPDH* 2) and ubiquitin (*UBI*), have been presented in Vuosku et al. (2009). For the *GAPDH* also a second primer pair (*GAPDH* 1) was utilized which generated a 94 bp long PCR product with following primers 5'-ACTAACTGCTTGGCCCCTT-3' (forward) and 5'-GTTGCTGTGATGGAATGCAC-3'(reverse). A dilution series of pooled cDNA samples was used to generate primer pair specific efficiencies of 1.9, 1.87 and 1.98 for *GAPDH* 1, *GAPDH* 2 and *UBI*, respectively. The qPCR runs were conducted as described in the Material and Methods. The Advanced Relative Quantification analysis of Lightcycler® 480 software release 1.5.0 SP3 was utilized to generate the positive calibrator normalized relative expression values (Roche Applied Science Technical Note No. LC 13/2001). The values present the ratio between the relative amount of target and reference genes normalized e.g. divided by the target/reference ratio of the positive calibrator (Roche Applied Science Technical Note No. LC 13/2001). The relative expression of each target gene was calculated using both reference genes and one biological replicate presenting early embryogeny was utilized as a positive calibrator.

The qPCR results presenting the expression of *CAT*, *RBR*, and *βG* in developing seeds at the early and late embryogeny and in the embryos and megagametophytes of mature seeds calculated using absolute quantification and positive calibrator normalized quantification are presented in Figure S1. The expression generated with two quantification methods was comparable, however, the deviation was greater when the expression was calculated using the reference genes (positive calibrator normalized relative expression procedure). Statistically significant differences were found in the *CAT* expression between early and late embryogeny and between embryos and megagametophytes of mature seeds. The statistical examination of data was performed with two sample t test and Wilcoxon rank sum test with a threshold of $P < 0.1$.

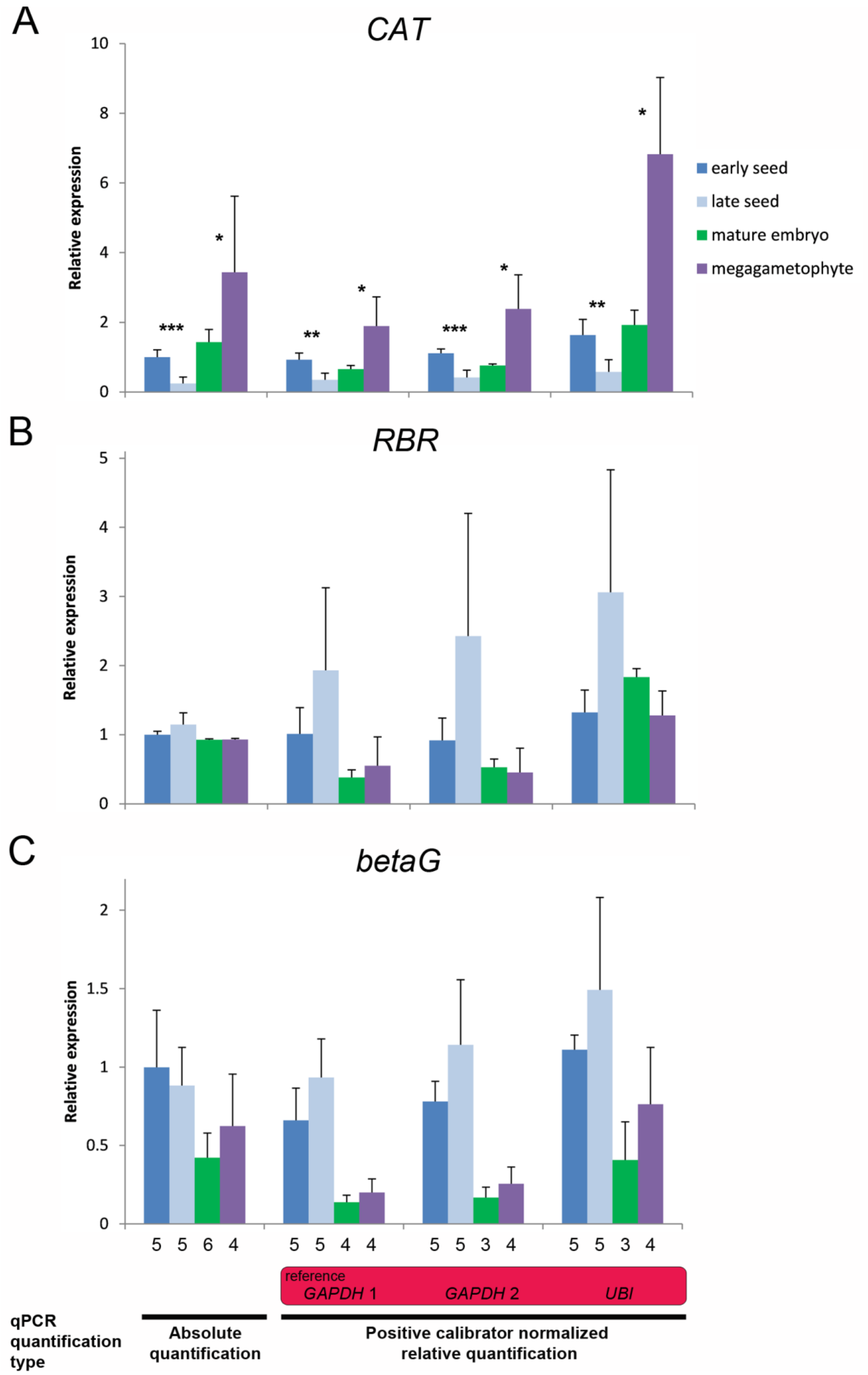

Figure S1. The expression (average + SD) of *CAT*, *RBR*, and *βG* calculated using absolute quantification and positive calibrator normalized quantification in developing seeds at the early and late embryogeny and in the embryos and megagametophytes of mature seeds. Number of biological replicates presented below bars. **P* < 0.1, ***P* < 0.01, ****P* < 0.001.
