# Supplementary material for: Expression of catalase and retinoblastoma-related protein genes associates with cell death processes in Scots pine zygotic embryogenesis
Source: BMC Plant Biol. 2015 Mar 15;15:88. doi: 10.1186/s12870-015-0462-0 (PMC4396594; doi:10.1186/s12870-015-0462-0)
Supplement: Additional file 3: — Detection of nuclear DNA fragmentation by TUNEL assay. [file 12870_2015_462_MOESM3_ESM.pdf]

Additional file 3

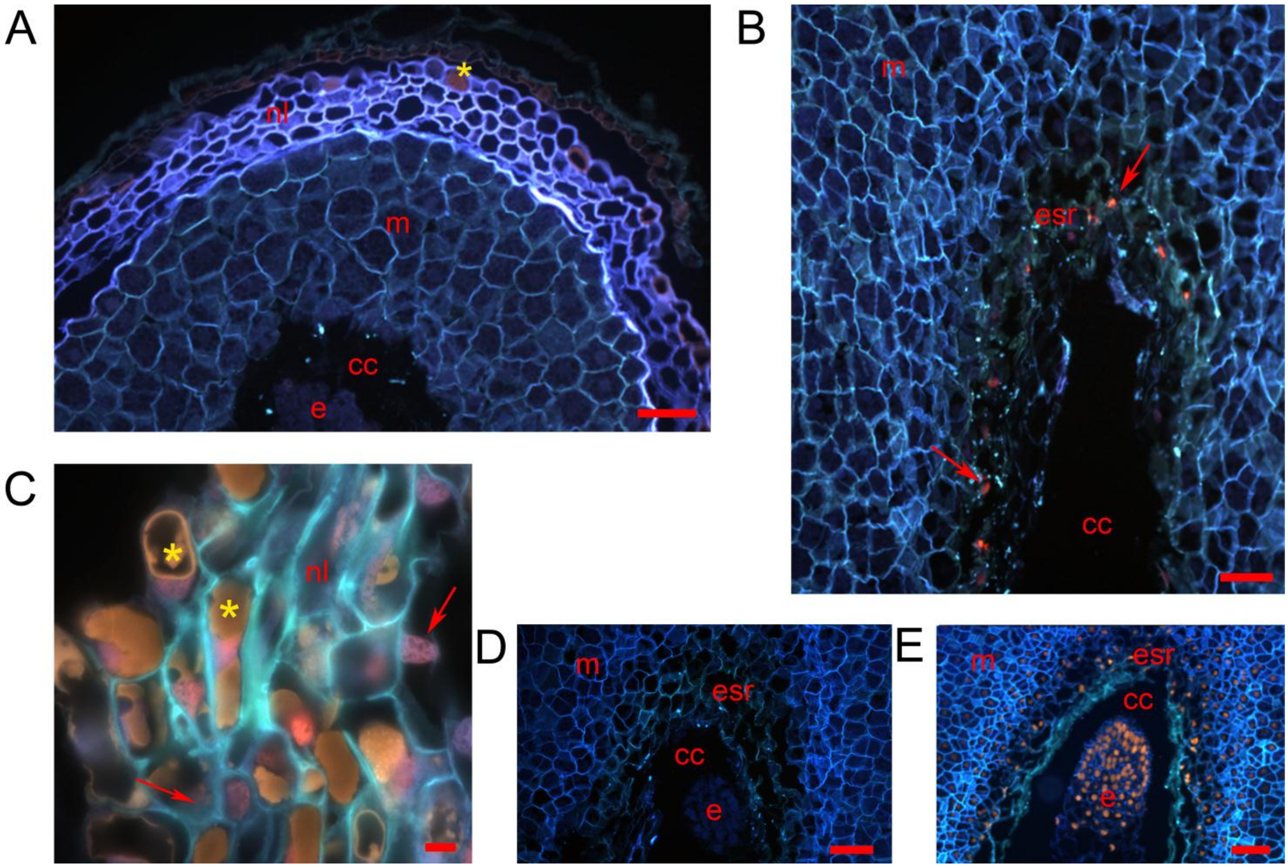

Figure S2. Nuclear DNA fragmentation in Scots pine seed section detected by TUNEL assay. (A) At the early embryogeny DNA fragmentation was not observed in the nucellar layers or megagametophyte cells apart from (B) cell of the ESR. (C) At late embryogenesis the TUNEL-positive signals were abundant in the nucellar layer.). (D) Negative control (omission of TdT). (E) Positive control (DNase treatment). cc=corrosion cavity, e=embryo, esr=embryo surrounding region, m=megagametophyte, nl=nucellar layers. Star, tannins; arrow, TUNEL-positive signals. Bars: (C) 10 µm, (A, B) 50 µm, and (D, E) 100 µm.
