# Supplementary material for: Expression of catalase and retinoblastoma-related protein genes associates with cell death processes in Scots pine zygotic embryogenesis
Source: BMC Plant Biol. 2015 Mar 15;15:88. doi: 10.1186/s12870-015-0462-0 (PMC4396594; doi:10.1186/s12870-015-0462-0)
Supplement: Additional file 4 — In situ mRNA hybridizations with sense probes. [file 12870_2015_462_MOESM4_ESM.pdf]

Additional file 4

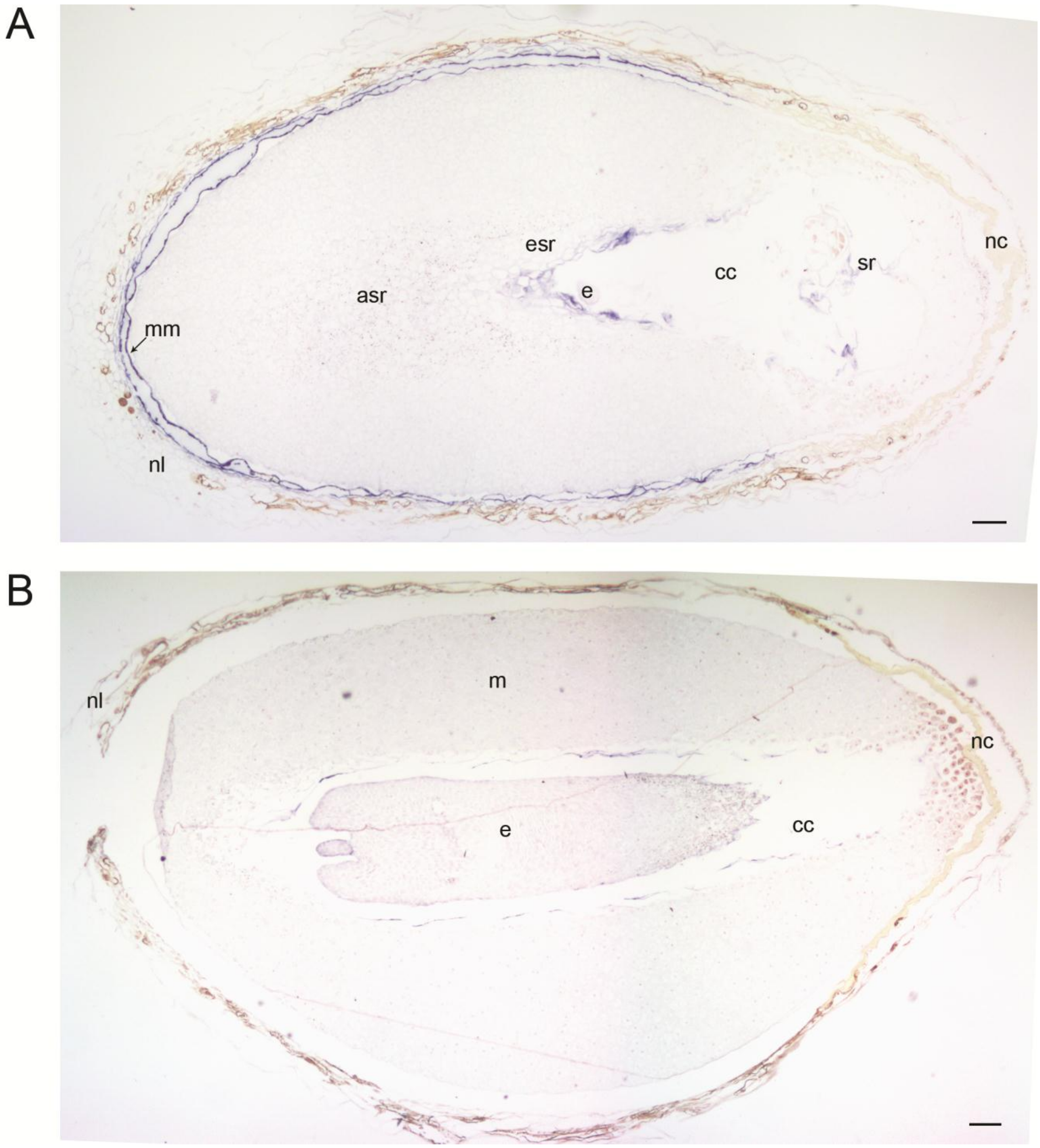

Figure S3. The sections hybridized with the sense *CAT* probe in a developing Scots pine seed at the early embryogeny (A) and at the late embryogeny (B). asr=arrow-shaped region, cc=corrosion cavity, e=embryo, esr=embryo surrounding region, m=megagametophyte, mm=megaspore membranes, nc=nucellar cap, nl=nucellar layers, sr=suspensor remnants. Bars: 100 µm.

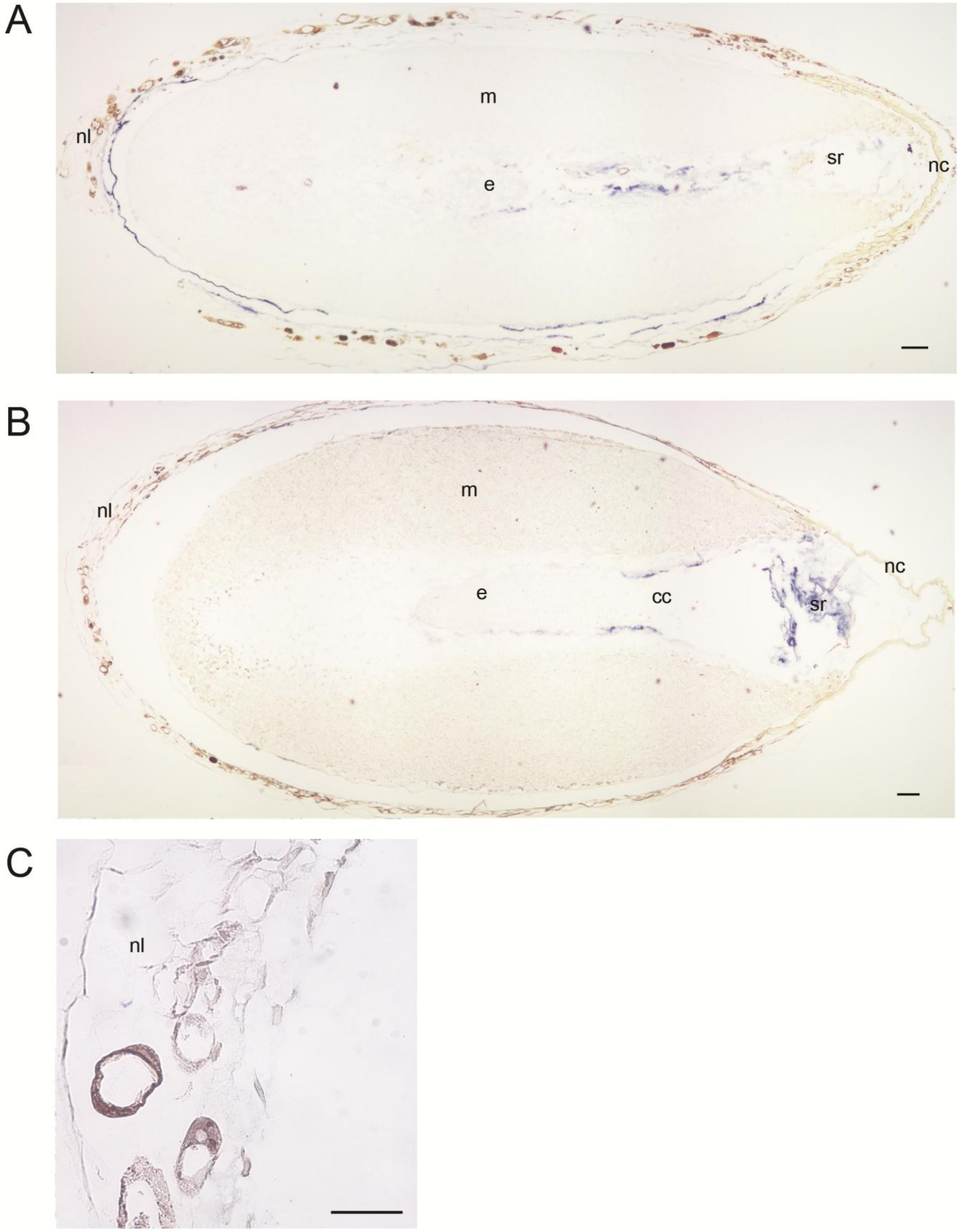

Figure S6. The sections hybridized with the sense *RBR* probe in a developing Scots pine seed at the early embryogeny (A) and at the late embryogeny (B). cc=corrosion cavity, e=embryo, m=megagametophyte, nc=nucellar cap, nl=nucellar layers, sr=suspensor remnants.Bars: 100 µm (A, B) and 20 µm (C).

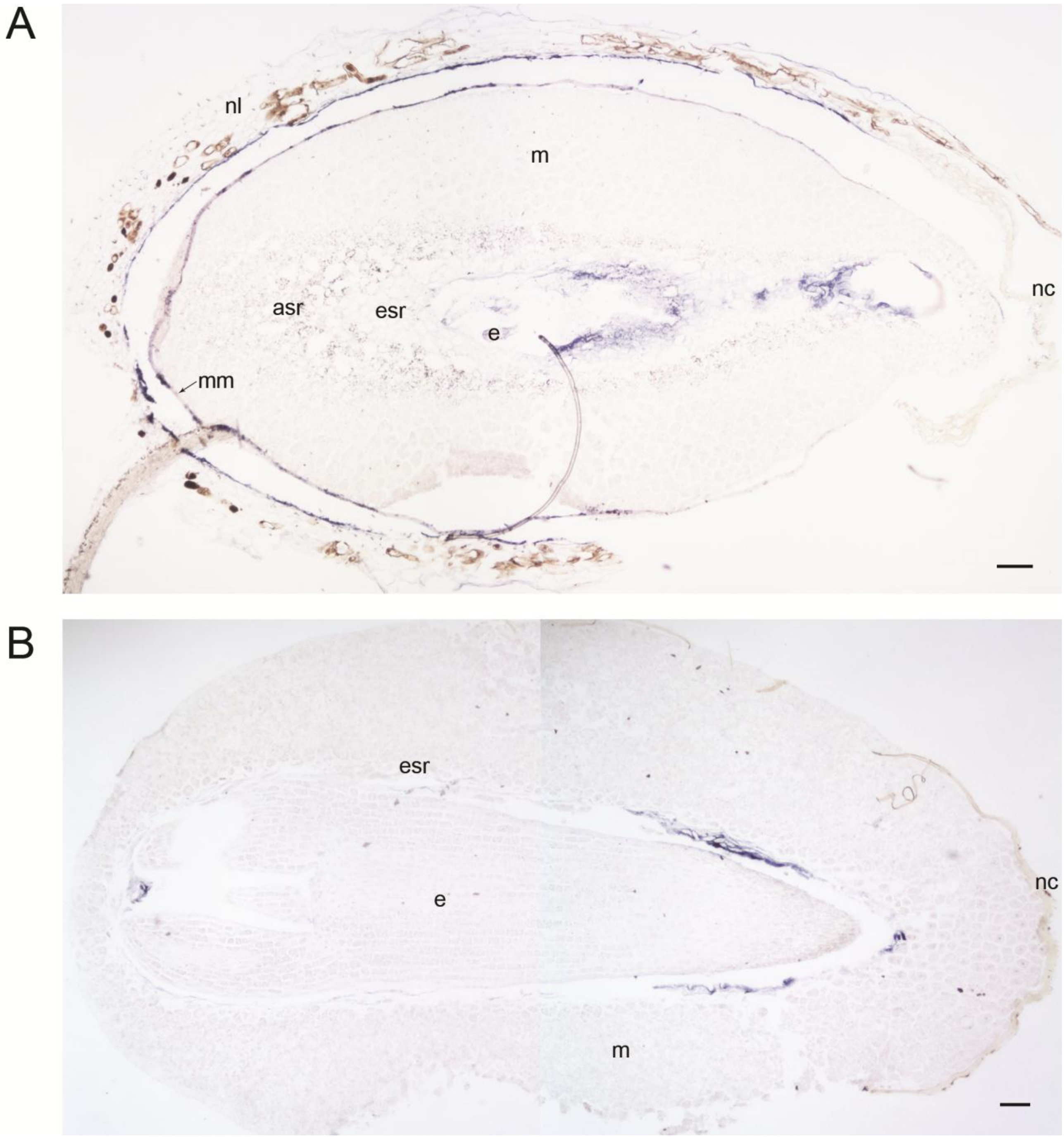

Figure S7. The sections hybridized with the sense *ATG5* probe in a developing Scots pine seed at the early embryogeny (A) and at the late embryogeny (B). asr=arrow-shaped region, e=embryo, esr=embryo surrounding region, m=megagametophyte, mm=megaspore membranes, nc=nucellar cap, nl=nucellar layers. Bars: 100 μm.
