# Supplementary material for: Expression of catalase and retinoblastoma-related protein genes associates with cell death processes in Scots pine zygotic embryogenesis
Source: BMC Plant Biol. 2015 Mar 15;15:88. doi: 10.1186/s12870-015-0462-0 (PMC4396594; doi:10.1186/s12870-015-0462-0)
Supplement: Additional file 5: — Verification of DAB visualized peroxidase location with H 2 O 2 supplemented TMB. [file 12870_2015_462_MOESM5_ESM.pdf]

Additional file 5

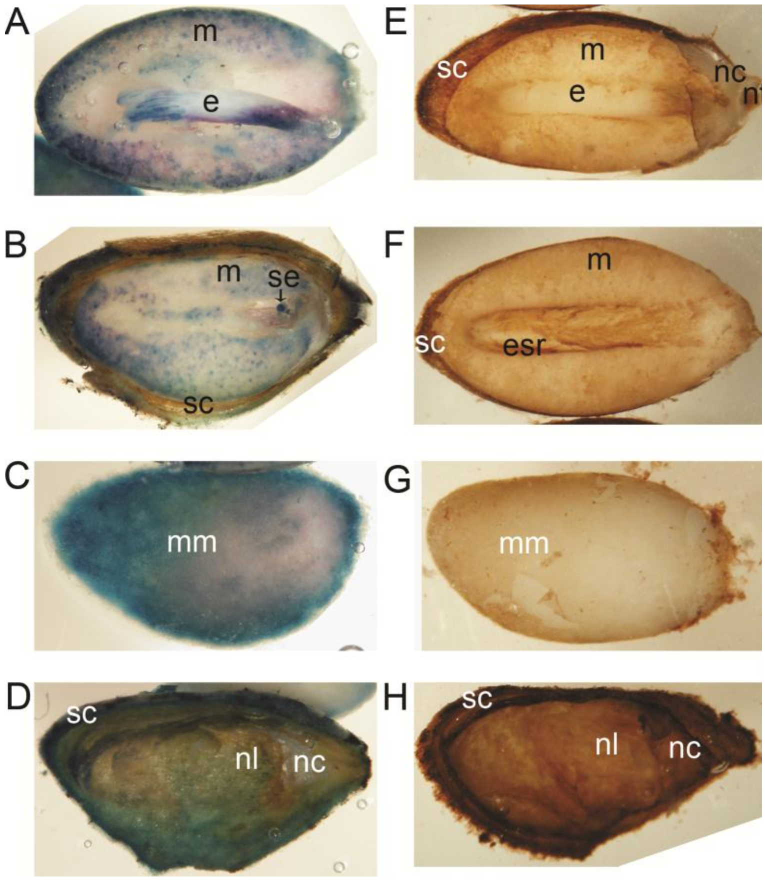

Figure S4. Verification of DAB visualized peroxidase location with H_2O_2 supplemented with TMB. (A-D) Peroxidase activity (blue colour) in Scots pine seeds visualized by using TMB supplemented with 3% H_2O_2. (E-H) Peroxidase activity (brown colour) in Scots pine seeds visualized by oxidized DAB. At the late embryogeny peroxidase was located in the (A, E) megagametophyte cells, (B) subordinate embryo, (B, F) in the loose megagametophyte cells surrounding corrosion cavity, (C,G) megaspore membranes, (D, H) seed coat and nucellar layers. e=embryo, esr=embryo surrounding region, m=megagametophyte, mm=megaspore membranes, nc=nucellar cap, nl=nucellar layers, nt=cellular nucellus, sc=seed coat, se=subordinate embryo, sr=suspensor remnants.
