# Supplementary material for: Expression of catalase and retinoblastoma-related protein genes associates with cell death processes in Scots pine zygotic embryogenesis
Source: BMC Plant Biol. 2015 Mar 15;15:88. doi: 10.1186/s12870-015-0462-0 (PMC4396594; doi:10.1186/s12870-015-0462-0)
Supplement: Additional file 6: — RBR localization with in situ mRNA hybridization in the transition from early to late developmental stage of the Scots pine seed development. [file 12870_2015_462_MOESM6_ESM.pdf]

Additional file 6

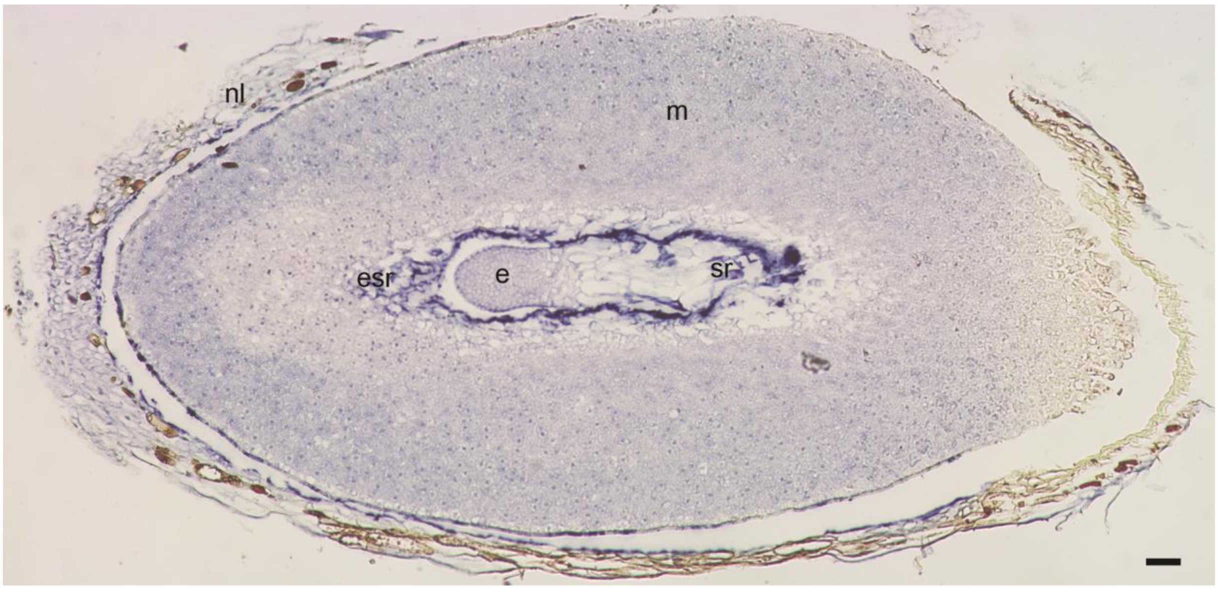

Figure S5. The localization of *RBR* mRNAs (blue signal) in a developing Scots pine seed. *RBR* expression in the megagametophyte, embryo surrounding region, embryo and nucellar layers. asr=arrow-shaped region, e=embryo, esr=embryo surrounding region, m=megagametophyte, nl=nucellar layers, sr=suspensor remnants. Bar: 100 µm.
