# Supplementary material for: Expression of catalase and retinoblastoma-related protein genes associates with cell death processes in Scots pine zygotic embryogenesis
Source: BMC Plant Biol. 2015 Mar 15;15:88. doi: 10.1186/s12870-015-0462-0 (PMC4396594; doi:10.1186/s12870-015-0462-0)
Supplement: Additional file 7: — βG expression in developing Scots pine seeds. [file 12870_2015_462_MOESM7_ESM.pdf]

Additional file 7

Glycoside hydrolases (GHs) are involved in the metabolism of various carbohydrates containing compounds present in plant tissues, but the majority of the GH enzymes are involved in cell wall polysaccharide metabolism (Cosgrove, 2005; Minic and Jouanin, 2006; Minic, 2008). In Arabidopsis (*Arabidopsis thaliana* L.), cell wall associated *β*-glucosidase (*β*G, EC 3.2.1.21), which breaks down polysaccharides to soluble sugars, is induced by starvation (Lee *et al.*, 2007) and senescence (Mohapatra *et al.*, 2010). In barley (*Hordeum vulgare* L.), hydrolysis of *β*-linked oligosaccharides results in cell wall degradation in endosperm during seed germination (Leah *et al.*, 1995).

For studying the role of cell wall hydrolyzing enzyme *β*G in the cell wall breakdown, the Scots pine *β*G gene (KM046994) was sequenced. The predicted *β*G protein shows 97% identity with the *β*G protein identified previously from lodgepole pine (*Pinus contorta* Dougl. var. latifolia Engelm.) xylem (Dharmawardhana *et al.*, 1995). No significant difference could be detected with the number of *βG* mRNA transcripts between early and late embryogenesis or the mature seeds and megagametophyte. However, the average *βG* mRNA transcripts levels showed somewhat similar trend to that observed with *CAT* transcripts, being higher in early embryogeny and in megagametophyte tissues than in late embryogeny or mature embryos, respectively (Figure S7A). During the early embryogeny, *βG* expressed strongly in the megagametophyte cells in the arrow shaped region in the front of the corrosion cavity preparing to die (Figure S7B, D). At the late embryogeny, *βG* expressed strongly in the cells of the nucellar layers (Figure S7F, G). The specificity of the antisense *βG* probe was confirmed by the absence of signals in the sections hybridized with the sense *βG* probe (Supplementary Figure S8).

Cosgrove DJ. 2005. Growth of the plant cell wall. *Nature Reviews Molecular Cell Biology* 6, 850-861.

Dharmawardhana *et al.*, 1995

Lee E, Matsumura Y, Soga K, Hoson T, Koizumi N. 2007. Glycosyl hydrolases of cell wall are induced by sugar starvation in arabidopsis. *Plant and Cell Physiology* 48, 405-413.

Minic Z. 2008. Physiological roles of plant glycoside hydrolases. *Planta* 227, 723-740.

Minic Z, Jouanin L. 2006. Plant glycoside hydrolases involved in cell wall polysaccharide degradation. *Plant Physiology and Biochemistry* 44, 435-449.

Mohapatra PK, Patro L, Raval MK, Ramaswamy NK, Biswal UC, Biswal B. 2010. Senescence-induced loss in photosynthesis enhances cell wall ß-glucosidase activity. *Physiologia Plantarum* 138, 346-355.

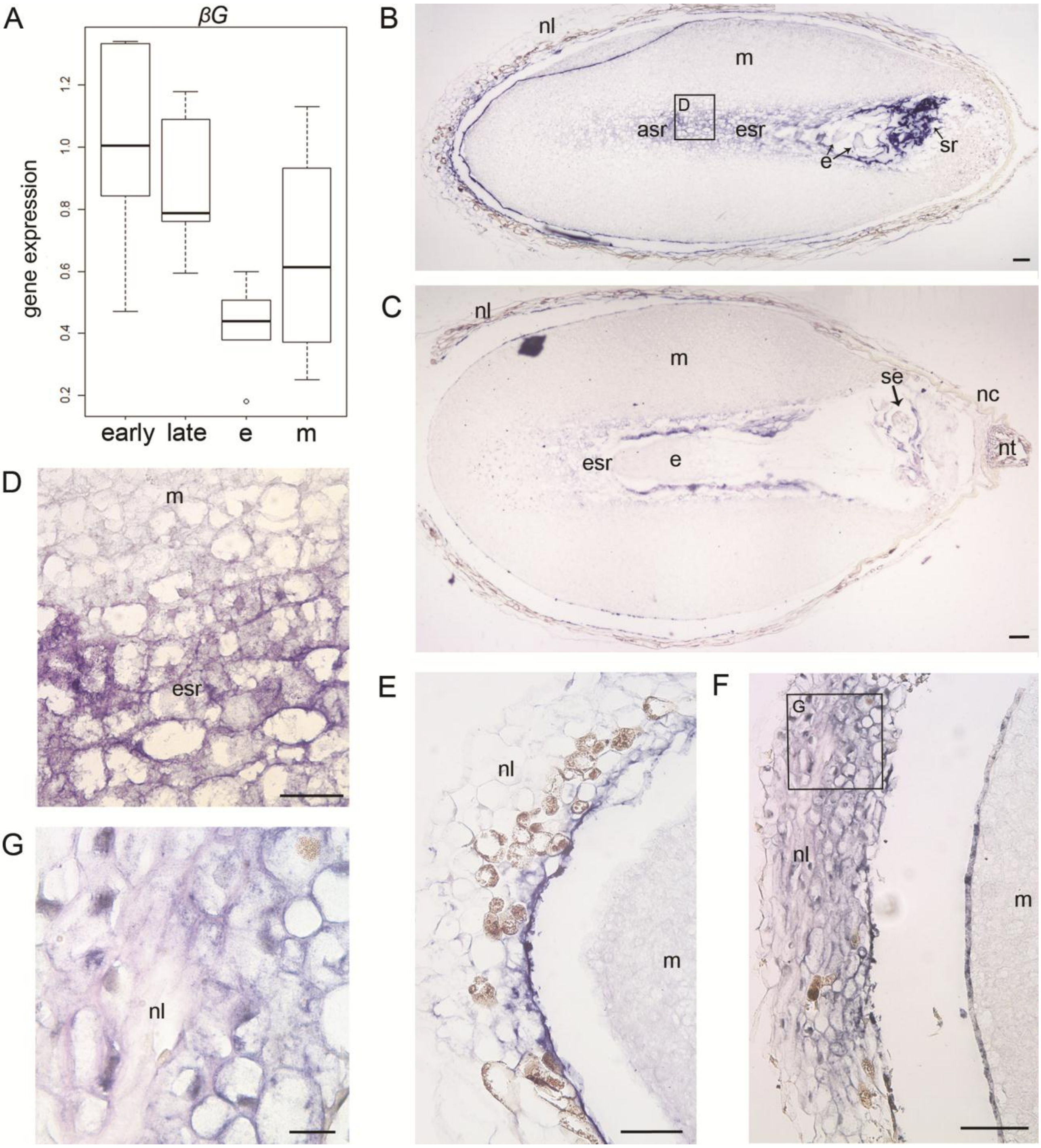

Figure S8. *βG* expression in developing Scots pine seeds. (A) The expression of *βG* in developing seeds at the early and late embryogeny and in the embryos (e) and megagametophytes (m) of mature seeds. The expression was based on mRNA copy numbers generated with the absolute Q-RT-PCR analysis and values presented were normalized using the expression at the early embryogeny. (B) The localization of *βG* mRNAs (blue signal) in a developing Scots pine seed at the early embryogeny. (C) The localization of *βG* mRNAs at the late embryogeny. (D) Intense *βG* expression in the megagametophyte cells in the ESR at the early embryogeny. (E) Weak *βG* expression in the cells of the nucellar layers at the early embryogeny. (F, G) Intense *βG* expression in the cells of the nucellar layers at the late embryogeny. asr=arrow-shaped region, cc=corrosion cavity, e=embryo, esr=embryo surrounding region, m=megagametophyte, nc=nucellar cap, nl=nucellar layers, nt=cellular nucellus, se=subordinate embryo, sr=suspensor remnants. Bars: (G) 20 µm, (D) 50 µm, and (B, C, E, F) 100 µm.

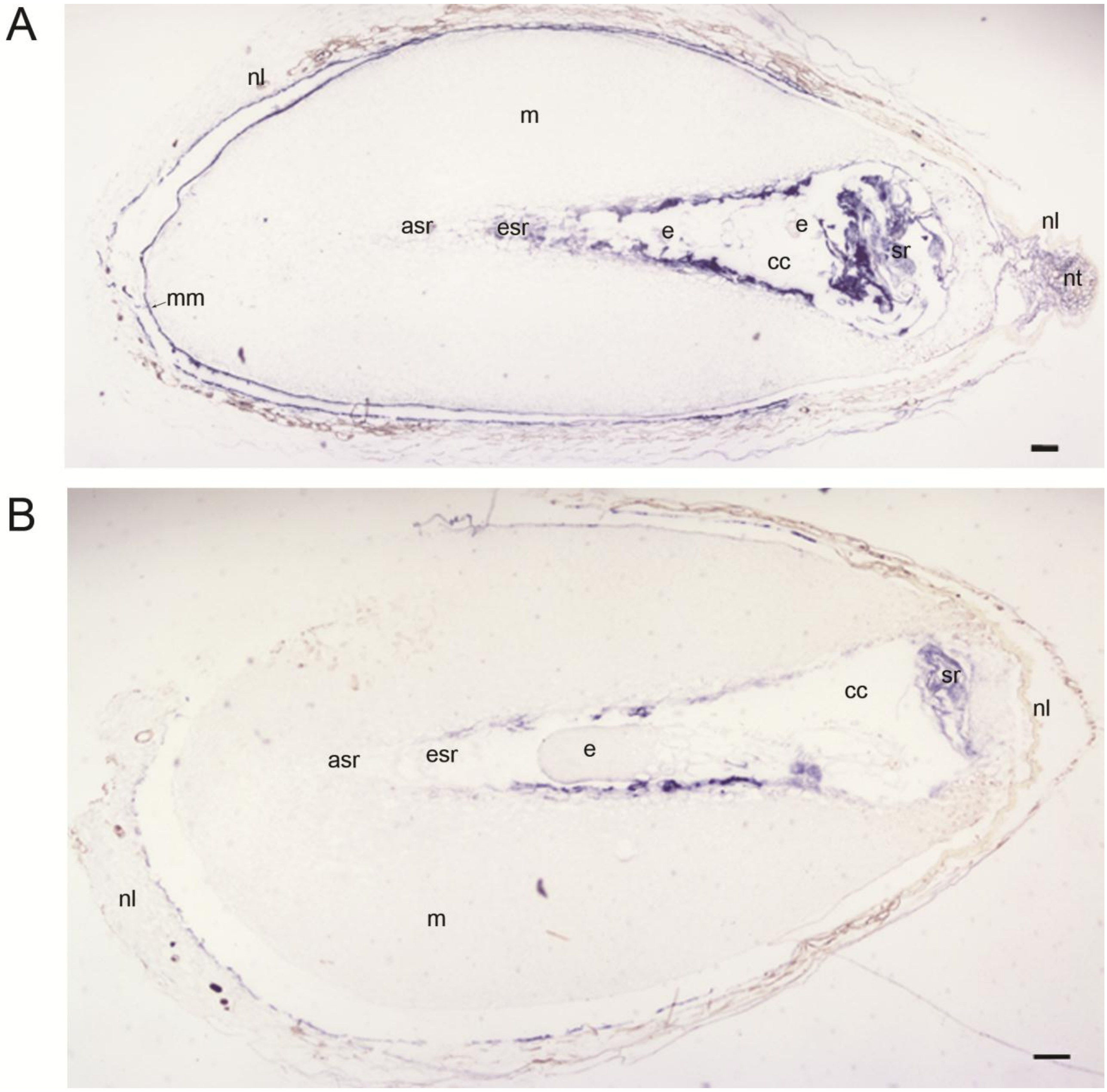

Figure S9. The sections hybridized with the sense *βG* probe in a developing Scots pine seed at the early embryogeny (A) and at the late embryogeny (B). asr=arrow-shaped region, cc=corrosion cavity, e=embryo, esr=embryo surrounding region, m=megagametophyte, mm=megaspore membranes, nc=nucellar cap, nl=nucellar layers, nt=cellular nucellus, sr=suspensor remnants. Bars: 100 μm.
